# Wunderlich Syndrome: A Rare Case of Spontaneous Perinephric Hemorrhage

**DOI:** 10.7759/cureus.107309

**Published:** 2026-04-18

**Authors:** Tutul Chowdhury, Aditi Parulkar, Anusha Akella, Nisha Sapkota, Mehak G Mastoi, Minhaz Murshad, Erum Zahid, Raymond Beyda

**Affiliations:** 1 Pulmonary and Critical Care Medicine, One Brooklyn Health, Interfaith Medical Center and Brookdale University Hospital, New York, USA; 2 Internal Medicine, One Brooklyn Health, Interfaith Medical Center, New York, USA; 3 Emergency and Critical Care Medicine, One Brooklyn Health, Interfaith Medical Center and Brookdale University Hospital, New York, USA

**Keywords:** complex renal cyst, end-stage renal disease (esrd), perinephric hemerrhage, renal hemotoma, retroperitoneal bleeding, spontaneous perinephric hemorrhage, wunderlich syndrome

## Abstract

Wunderlich syndrome is a rare urological emergency characterized by spontaneous, nontraumatic renal hemorrhage into the subcapsular and perirenal spaces. We present the case of a 37-year-old male with end-stage renal disease on hemodialysis, hypertension, and bilateral inguinal hernias who presented with acute chest and abdominal pain, nausea, and vomiting after missing a dialysis session. The initial assessment identified severe anemia necessitating a massive transfusion, and he was subsequently treated for suspected uremic coagulopathy. Imaging with computed tomography angiography (CTA) demonstrated a massive left retroperitoneal hemorrhage. The patient also tested positive for COVID-19, potentially contributing to a complex coagulopathic state. Given ongoing bleeding, he underwent successful renal artery embolization, achieving hemostasis without surgical intervention. This case highlights the importance of early recognition of Wunderlich syndrome in high-risk patients, the diagnostic value of CTA, and the critical role of interventional radiology in management.

## Introduction

Wunderlich syndrome is a rare, life-threatening condition defined by spontaneous, nontraumatic renal hemorrhage into the subcapsular and perirenal spaces. Although classically associated with Lenk’s triad of flank pain, palpable mass, and shock, presentations are often nonspecific, leading to delayed diagnosis [1]. Common causes include renal neoplasms and ruptured renal cysts, while coagulopathies and vascular abnormalities are increasingly recognized. In addition to uremic platelet dysfunction, patients with end-stage renal disease (ESRD) on hemodialysis who receive heparin are at increased risk of such complications, and these patients are also prone to developing acquired cystic kidney disease, which may pose another risk [1, 2]. Early diagnosis with computed tomography angiography (CTA) is crucial for prompt management [3, 4]. We present a case highlighting the diagnostic challenges and importance of timely intervention in Wunderlich syndrome.

## Case presentation

A 37-year-old male with ESRD secondary to hypertensive nephropathy, on maintenance hemodialysis, presented with acute-onset abdominal pain and shortness of breath, along with multiple episodes of nonbloody, nonbilious vomiting after missing several hemodialysis sessions prior to admission. His medical history was significant for hypertension, HFpEF, pulmonary hypertension, and anemia of chronic disease. Physical examination revealed abdominal distension and diffuse tenderness. On arrival, blood pressure was 110/70 with a MAP of 72, and heart rate was 90. Laboratory evaluation was notable for hyperkalemia and an initial hemoglobin of 6.9 g/dL (baseline hemoglobin was 11 g/dL). Laboratory investigation results on admission are given in Table 1. 

**Table 1:** Laboratory investigation results on admission PCR: polymerase chain reaction.

Investigation	On admission	Day 7	Day 14	References range
Hemoglobin	5.7	6.5	9.1	11.0-15.0 g/dL
Hematocrit	16.4	19.2	27.3	35-46%
White blood cell	6.3	6.5	9.4	3.8-5.3 × 10^6^/mL
Platelets	218	169	245	130-400 × 10^3^/mL
Troponin	97	55	83	5.0-19.7 pg/mL
Glucose	94	110	137	80-115 mg/dL
Blood urea nitrogen	97	46	41	9.8-20.1 mg/dL
Creatinine	10.3	6.7	5.1	0.57-1.11 mg/dL
Sodium	134	135	134	136-145 mmol/L
Potassium	5.8	4.9	4.2	3.5-5.1 mmol/L
Chloride	97	94	96	98-107 mmol/L
Bicarbonate	24	26	25	23-31 mmol/L
Calcium	8.7	7.8	8.3	8.8-10.0 mg/dL
Albumin	3.7	3.3	3.4	3.2-4.6 g/dL
Magnesium	2.0	1.8	2.1	1.6-2.6 mg/dL
COVID PCR	Positive			Negative
Prothrombin time	14.4	13.7	13.1	9.8-13.4 seconds
International Normalized Ratio (INR)	1.18	1.26	1.21	0.85-1.15
Partial thromboplastin time (PTT)	36.2	38.5	35.1	24.9-35.9 seconds
Thyroid-stimulating hormone	2.41			0.465-4.680 IU/mL
Urine toxicology	Negative			Negative

**Figure 1:**
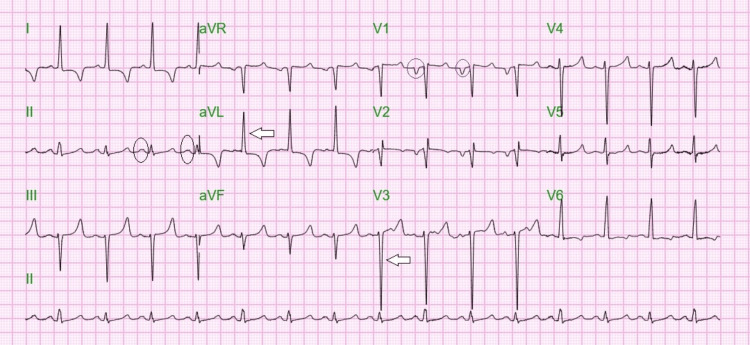
Electrocardiogram showing broad, bifid P wave or P mitrale in lead II (black circles) and total length of >28 millimeters R wave in aVL + S-wave in V3 as per Cornell Voltage Criteria (white arrows)

EKG on admission showed left atrial enlargement and left ventricular hypertrophy (Figure 1). He demonstrated persistent severe anemia despite repeated transfusions five times; later, he developed progressive abdominal pain, distension, and groin tenderness. 

**Figure 2:**
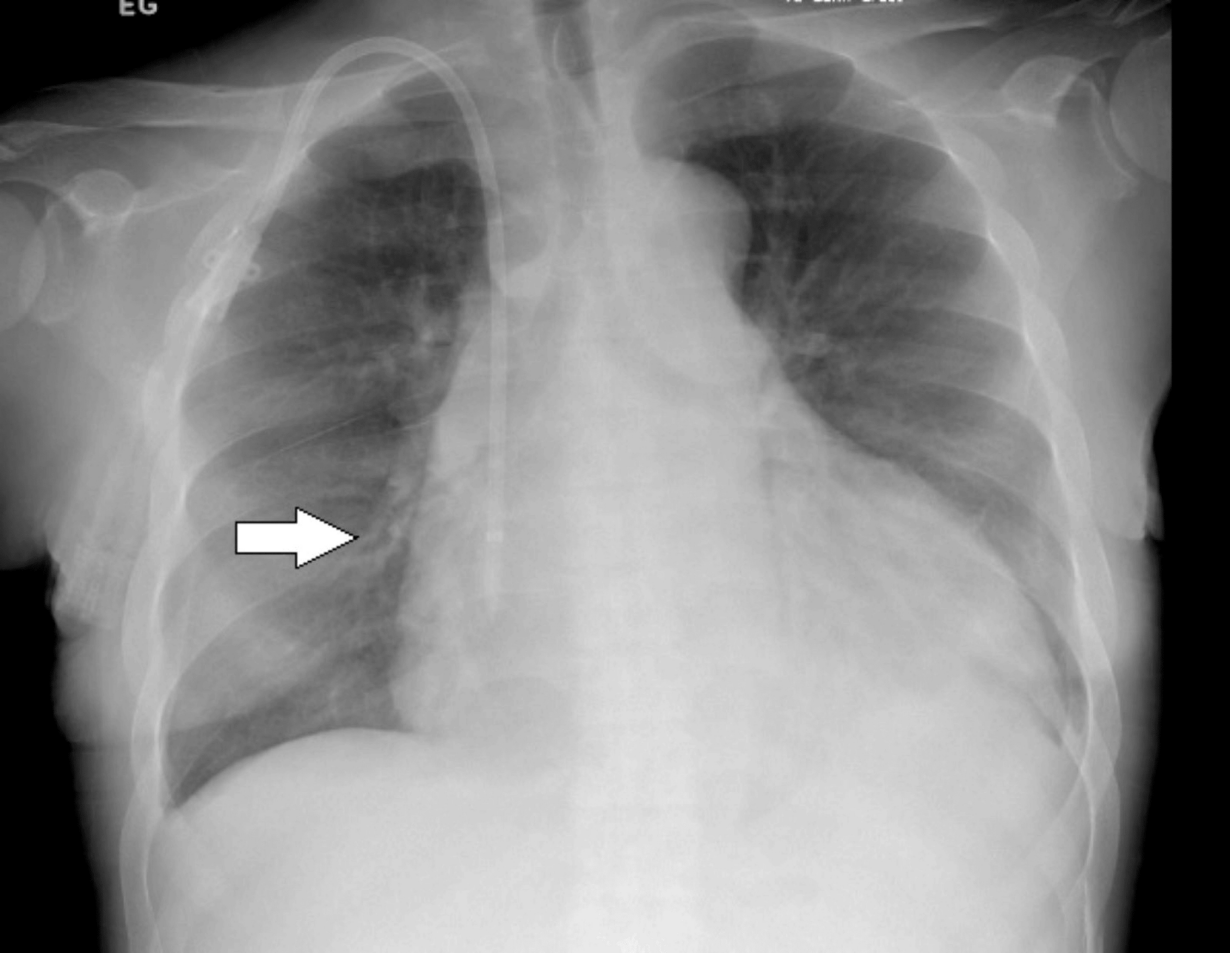
Chest X-ray showing vascular congestion (white arrow)

A chest X-ray showed findings consistent with pulmonary vascular congestion (Figure 2).

**Figure 3:**
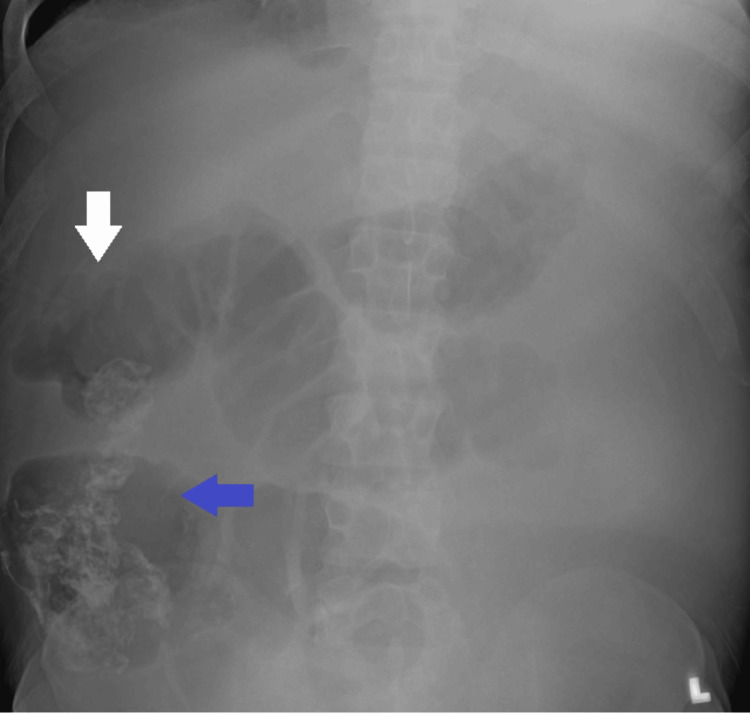
X-ray abdomen showed distention of the cecum (blue arrow) and distension of ascending colon (white arrow)

X-ray abdomen showed distention of the cecum and distension of the ascending colon (Figure 3).

**Figure 4:**
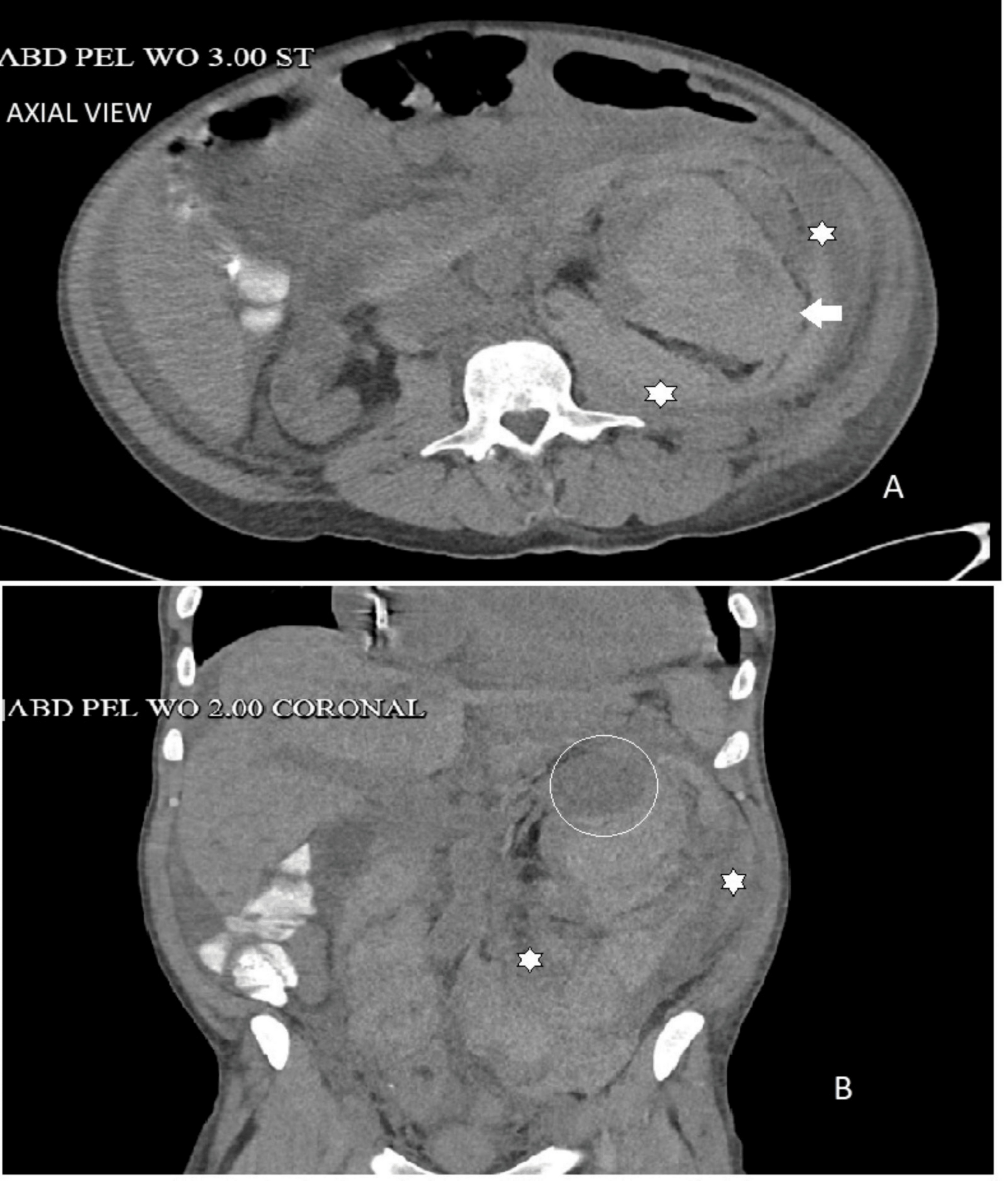
Computed tomography angiography showing the left kidney (white arrow) with extensive perinephric and retroperitoneal blood (asterisk marks in image A, axial view, and image B, coronal view), with a renal cyst (white circle) in image B

The patient concurrently tested positive for COVID-19 and, given his mild disease symptoms, was initiated on a five-day course of Paxlovid. To address potential coagulopathy, he was treated with desmopressin (renal-adjusted dose), tranexamic acid, and conjugated estrogen injections. Further investigation into the abdominal pain and severe anemia included CTA of the chest, abdomen, and pelvis. This imaging study was critical, revealing a massive left retroperitoneal hemorrhage directly linked to a high-grade injury (American Association for the Surgery of Trauma (AAST) grade 4/5) to the left kidney, with possible involvement of the vascular pedicle (Figure 4). 

**Figure 5:**
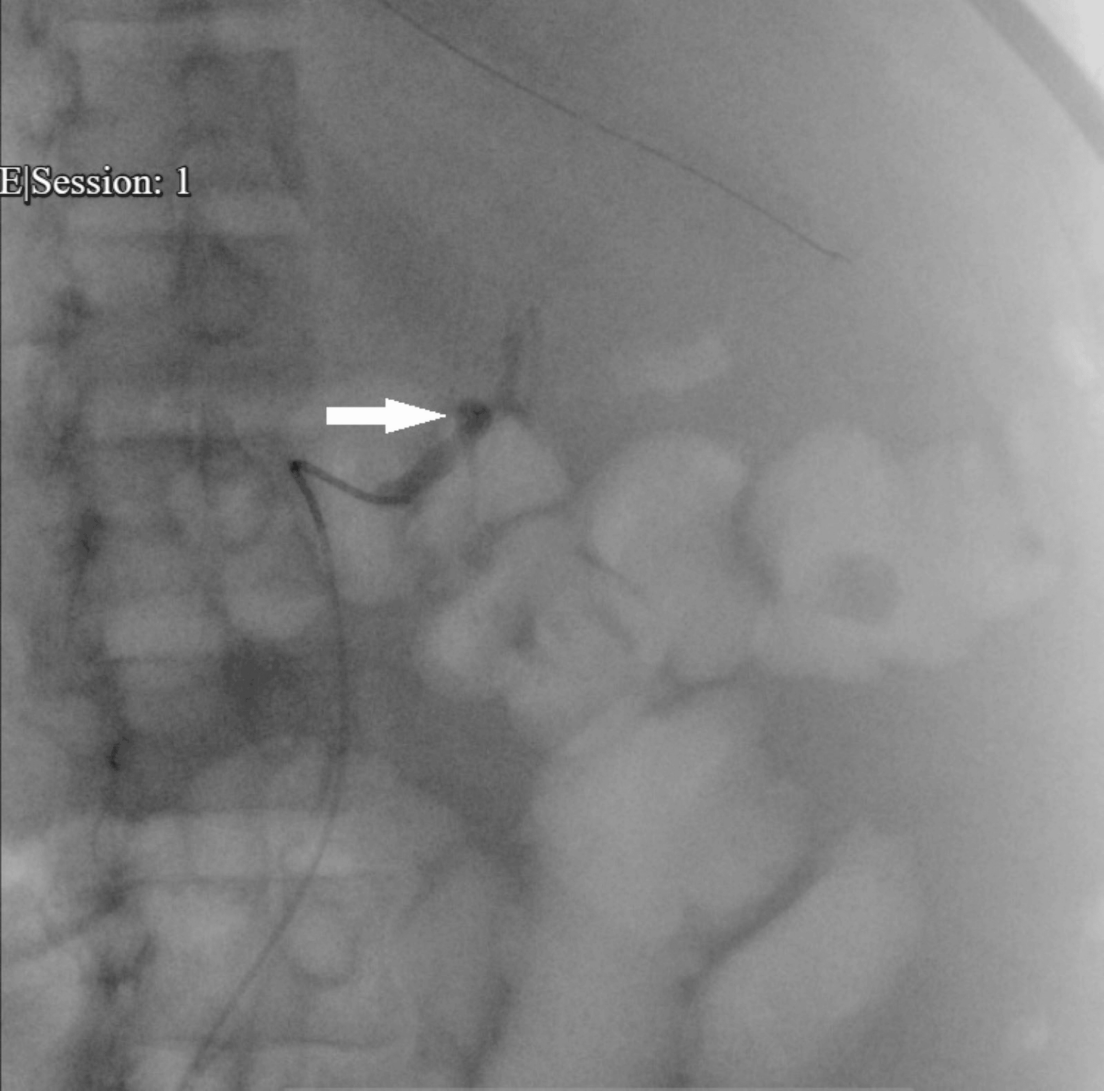
Left renal arteriogram showing pseudoaneurysm

A renal ultrasound of the right kidney showed a size of 11.0 cm, with no hydronephrosis, but noted increased parenchymal echogenicity and multiple small cysts. The right renal findings were consistent with chronic kidney disease but did not show acute pathology. Due to ongoing bleeding and hemodynamic instability, the patient underwent IR-guided embolization of both left renal arteries, avoiding emergency radical nephrectomy (Figure 5). A left renal arteriogram revealed a pseudoaneurysm (Figure 5). He required a total of 14 units of packed red blood cells (PRBCs) prior to the procedure. Post-embolization, the patient was initiated on continuous renal replacement therapy (CRRT) for volume management and resistant hypertension, eventually transitioning back to a three-times-a-week intermittent hemodialysis schedule. Severe abdominal pain and distension necessitated nasogastric (NG) tube decompression and a multimodal analgesic regimen, including a fentanyl patch and intravenous opioids. Following embolization, his hemoglobin stabilized above 8 g/dL, and serial imaging confirmed no further expansion of the hematoma. At the time of discharge, his pain was well controlled, and his volume status was stable. He was discharged to a subacute rehabilitation facility for physical and occupational therapy due to deconditioning, with planned outpatient follow-up. 

## Discussion

Wunderlich syndrome, defined as spontaneous nontraumatic renal hemorrhage into the subcapsular and perirenal spaces, remains a rare but life-threatening clinical entity. It is classically characterized by Lenk’s triad of acute flank pain, a palpable mass, and hypovolemic shock; however, this triad is present in only a minority of cases, often making early diagnosis challenging. In the present case, the patient’s initial presentation with chest pain, abdominal pain, nausea, and vomiting, in conjunction with profound anemia, highlights the nonspecific and potentially misleading clinical manifestations of this condition [1]. The etiology of Wunderlich syndrome is diverse, with renal neoplasms, particularly angiomyolipomas and renal cell carcinoma, being the most commonly reported causes [1, 2]. However, nonneoplastic etiologies such as vascular diseases, infections, and coagulopathies are increasingly recognized. In this patient, several risk factors likely contributed synergistically to the development of spontaneous retroperitoneal hemorrhage. ESRD is associated with uremic platelet dysfunction, which predisposes patients to bleeding despite normal platelet counts. Additionally, the missed hemodialysis session may have exacerbated uremia, further impairing hemostasis. Cases of Wunderlich syndrome have been reported in the literature in the setting of ESRD. The presence of severe anemia at presentation suggests ongoing or subacute bleeding prior to hospital arrival. The patient had several renal cysts; hence, cyst rupture was also considered a probable etiology in this case. The role of COVID-19 infection in this case is also noteworthy. Although COVID-19 is more commonly associated with thrombotic complications, there is growing evidence that it can also predispose to bleeding through mechanisms such as endothelial dysfunction, inflammation, and consumption of coagulation factors [3, 4]. This dual coagulopathic state may have compounded the patient’s underlying bleeding risk. Furthermore, the administration of multiple agents aimed at correcting uremic coagulopathy, including desmopressin, tranexamic acid, and conjugated estrogens, reflects the complexity of managing bleeding in ESRD patients, although their efficacy in the setting of massive retroperitoneal hemorrhage remains variable [5].

Imaging is essential in diagnosing Wunderlich syndrome, with CTA serving as the gold standard because it confirms the presence of hemorrhage while identifying its source and extent [5]. In this case, CTA revealed a massive left retroperitoneal hemorrhage with a high-grade (AAST IV/V) renal injury and possible vascular pedicle involvement, findings associated with increased morbidity and the need for urgent intervention.

Management is guided by hemodynamic status and bleeding etiology. Stable patients may be managed conservatively, whereas those with ongoing hemorrhage or instability require urgent intervention. Selective arterial embolization is now a preferred first-line treatment due to its minimally invasive approach, high success in achieving hemostasis, and potential for renal preservation [6, 7]. It is associated with lower morbidity and mortality compared to surgical exploration, especially in high-risk patients with multiple comorbidities. This case highlights key clinical considerations. Wunderlich syndrome should be suspected in patients with unexplained acute anemia and abdominal pain, particularly in the setting of ESRD or coagulopathy. Early use of CTA is essential for prompt diagnosis and management. Additionally, the combined effects of uremic platelet dysfunction and COVID-19-related coagulopathy may increase the risk of spontaneous hemorrhage. Interventional radiology remains central to the management of severe cases, providing effective and less invasive treatment options [3, 8, 9].

## Conclusions

In conclusion, Wunderlich syndrome is a rare but life-threatening condition that requires a high index of suspicion, particularly in patients presenting with unexplained acute anemia and abdominal pain. This case illustrates an atypical presentation in a patient with ESRD, in whom nonspecific symptoms initially obscured the diagnosis. The combination of uremic platelet dysfunction, missed hemodialysis, and concurrent COVID-19 infection likely contributed to an increased bleeding risk. Early use of CTA was critical in establishing the diagnosis and assessing the severity of renal injury. Prompt intervention with transarterial embolization successfully achieved hemostasis and avoided the need for surgical exploration. This case further emphasizes the growing role of interventional radiology as a first-line therapeutic approach in managing severe retroperitoneal hemorrhage. Overall, timely recognition and a multidisciplinary approach are essential to improving outcomes in patients with Wunderlich syndrome.
